# A miniaturized feedstocks-to-fuels pipeline for screening the efficiency of deconstruction and microbial conversion of lignocellulosic biomass

**DOI:** 10.1371/journal.pone.0305336

**Published:** 2024-10-08

**Authors:** Venkataramana R. Pidatala, Mengziang Lei, Hemant Choudhary, Christopher J. Petzold, Hector Garcia Martin, Blake A. Simmons, John M. Gladden, Alberto Rodriguez

**Affiliations:** 1 Joint BioEnergy Institute, Emeryville, CA, United States of America; 2 Biological Systems and Engineering Division, Lawrence Berkeley National Laboratory, Berkeley, CA, United States of America; 3 Department of Molecular and Cell Biology, University of California, Berkeley, Berkeley, CA, United States of America; 4 Department of Bioresource and Environmental Security, Sandia National Laboratories, Livermore, CA, United States of America; 5 Department of Biomaterials and Biomanufacturing, Sandia National Laboratories, Livermore, CA, United States of America; Government College University Faisalabad, PAKISTAN

## Abstract

Sustainably grown biomass is a promising alternative to produce fuels and chemicals and reduce the dependency on fossil energy sources. However, the efficient conversion of lignocellulosic biomass into biofuels and bioproducts often requires extensive testing of components and reaction conditions used in the pretreatment, saccharification, and bioconversion steps. This restriction can result in a significant and unwieldy number of combinations of biomass types, solvents, microbial strains, and operational parameters that need to be characterized, turning these efforts into a daunting and time-consuming task. Here we developed a high-throughput feedstocks-to-fuels screening platform to address these challenges. The result is a miniaturized semi-automated platform that leverages the capabilities of a solid handling robot, a liquid handling robot, analytical instruments, and a centralized data repository, adapted to operate as an ionic-liquid-based biomass conversion pipeline. The pipeline was tested by using sorghum as feedstock, the biocompatible ionic liquid cholinium phosphate as pretreatment solvent, a “one-pot” process configuration that does not require ionic liquid removal after pretreatment, and an engineered strain of the yeast *Rhodosporidium toruloides* that produces the jet-fuel precursor bisabolene as a conversion microbe. By the simultaneous processing of 48 samples, we show that this configuration and reaction conditions result in sugar yields (~70%) and bisabolene titers (~1500 mg/L) that are comparable to the efficiencies observed at larger scales but require only a fraction of the time. We expect that this Feedstocks-to-Fuels pipeline will become an effective tool to screen thousands of bioenergy crop and feedstock samples and assist process optimization efforts and the development of predictive deconstruction approaches.

## Introduction

The use of lignocellulosic biomass as feedstock for production of biofuels and bioproducts is a renewable alternative to petroleum-derived products since it represents the largest bioenergy reservoir on earth [1, 2]. Further, lignocellulosic biomass largely comprises human-inedible parts of plants that do not compete with human food supplies, attenuating concerns about land use and sustainability of crops and promoting the valorization of agricultural and forestry wastes [3, 4].

Despite its advantages, the conversion of biomass to biofuels and bioproducts in a predictable, scalable, and cost-efficient way remains a major challenge [5]. From a conversion perspective, this includes the need for a pretreatment step to increase the digestibility of biomass, which typically results in higher operational costs and has prompted the search for inexpensive, more effective, and easily recyclable pretreatment solvents [6]. Another important concern is the toxicity of the generated hydrolysates and produced fuels on the conversion microbes, which may require additional detoxification steps and strain engineering or screening efforts [7].

Environmental factors such as temperature and precipitation or the use of genetic engineering to develop feedstocks with beneficial traits can also play an important role during crop growth and influence the carbon storage mechanisms in plants and the resulting biomass composition [8]. These effects combined with the inherent heterogeneity of biomass has made it difficult to develop feedstock-agnostic processes and predictive deconstruction models, maintaining the requirement for individual testing of different biomass types and process parameters [9–11]. It is worth noting that not all of these studies include an assessment of sugar and biofuel conversion yields as performance parameters, which can be attributed to the lack of established high-throughput platforms covering the entire conversion process. Studying how different factors can impact the yields of sugars and fuels in a comprehensive way requires the characterization of a substantial number of samples, for which experiments at bench-scale level may be too time consuming [12].

High-throughput (HTP) pipelines used in biotechnology are automated systems that allow for the rapid screening of large numbers of microorganisms, enzymes or molecules [13]. Despite their advantages, previous efforts to apply HTP pipelines to improve the conversion of feedstocks to biofuels have been restricted to only certain stages of the process, which limits their applicability [14]. There is a need for a pipeline that comprises all steps in the process, is compatible with a one-pot configuration to reduce unit operations, and facilitates process integration, optimization and discovery work with minimal time-consuming solid-liquid separation steps.

Ionic liquids (ILs) are molten salts that have proven to be effective solvents for deconstruction of lignocellulosic biomass [15]. Some alkaline ILs such as cholinium lysinate are compatible with a one-pot process configuration that includes performing pretreatment, enzymatic hydrolysis and microbial conversion in the presence of the IL [16]. The IL cholinium phosphate has a less alkaline pH (thereby requiring less pH adjustment after pretreatment) but similar biocompatibility properties [17] to other cholinium-based ILs, making it better suited for a miniaturized Feedstocks-to-Fuels (F2F) HTP pipeline.

Here we report a laboratory-scale HTP pipeline that involves biomass weighing, pretreatment, enzymatic hydrolysis, fermentation, and metabolite analysis. An initial assessment of its performance and reproducibility was carried out using a representative feedstock (sorghum), IL (cholinium phosphate), enzymes (cellulase/hemicellulase cocktails), reaction conditions, and cultivation of an engineered strain of the yeast *Rhodosporidium toruloides* that produces the biofuel precursor bisabolene. This is the first report of the use of cholinium phosphate to perform a one-pot pretreatment, enzymatic hydrolysis, and microbial conversion process of a bioenergy feedstock such as sorghum. The purpose of the pipeline in its current form is not to produce the highest conversion yields but to allow for screening and characterization of the effects of using different variables. We expect that this platform will contribute to the rapid screening of genetic, environmental or process parameters used for converting biomass to biofuels and enable the development of computational models for performance predictions from the generated datasets.

## Materials and methods

**Fig 1:**
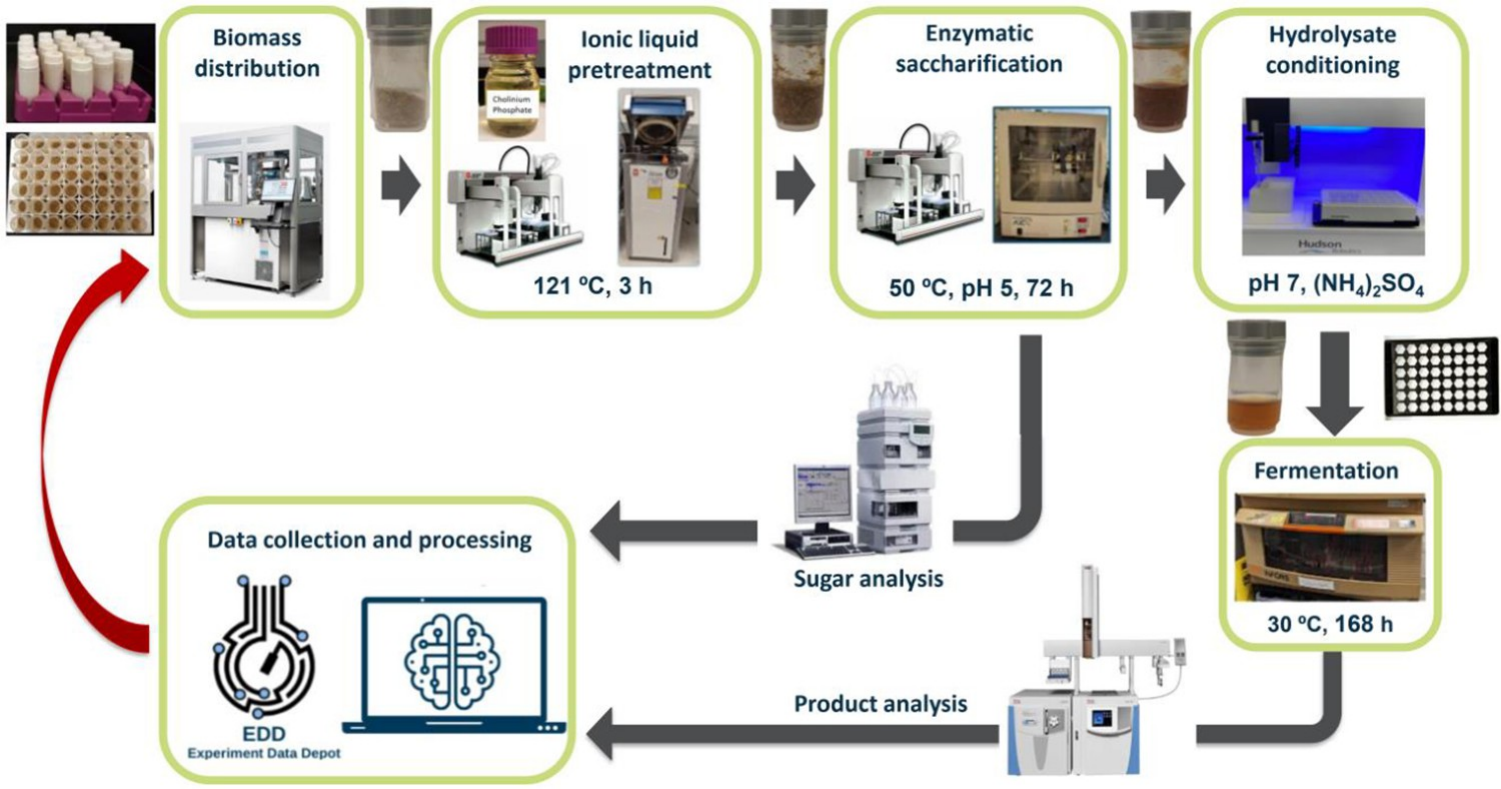
Overview of the feedstocks-to-fuels pipeline. The workflow begins with biomass weighing and distribution (top left) and ends with data collection and processing (bottom left). The processed data can be used in future campaigns to inform process parameter selection (depicted by the red arrow).

The protocol described in this peer-review article is published on protocols.io (https://dx.doi.org/10.17504/protocols.io.3byl4jrezlo5/v1) and is included for printing with this article (S1 File). Briefly, the methodological approach comprised the following steps: a) biomass weighing and distribution into plastic vials; b) addition of the ionic liquid cholinium phosphate to perform pretreatment using an autoclave; c) pH adjustment with citrate buffer, addition of cellulase and hemicellulase enzymes and incubation to degrade polysaccharides into fermentable sugars; d) filtration of hydrolysates and pH adjustment with phosphate buffer; e) cultivation of an engineered *R*. *toruloides* strain in the generated hydrolysates to convert sugars to bisabolene; and f) use of HPLC and GC-MS analytical methods to quantify glucose, xylose and bisabolene concentrations and perform data processing and analysis. The experimental workflow of the assembled F2F pipeline is outlined in Fig 1.

### Expected results

#### Biomass weighing and distribution

**Fig 2:**
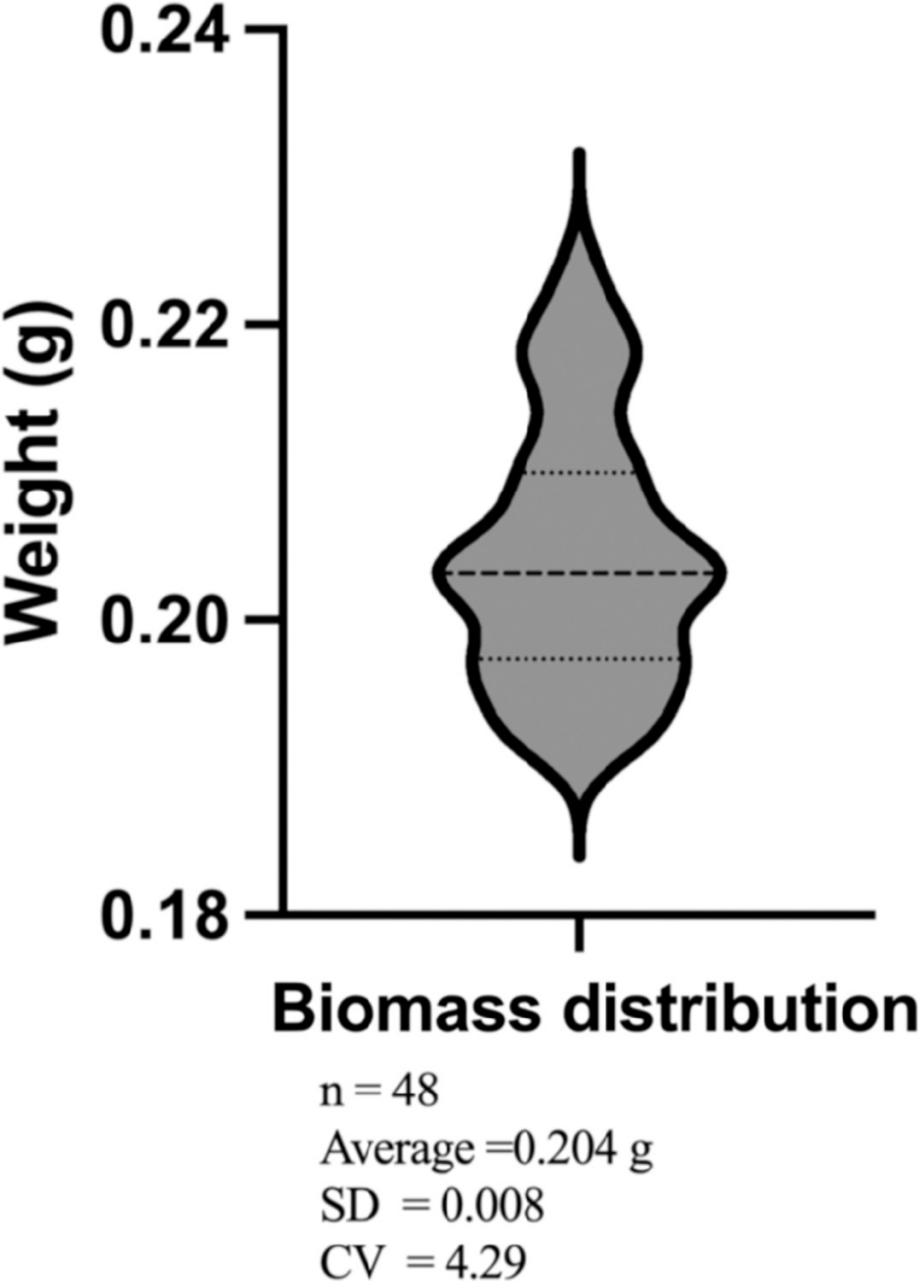
Biomass weight distribution using the Labman robot. A target weight of 0.225 g was used for the processing of 48 samples.

To validate the efficacy and reproducibility of the pipeline, we performed a complete conversion cycle by the simultaneous processing of 48 vials using wild type sorghum biomass as feedstock. The process begins with the distribution of dry biomass via a Labman solid handling robot (Labman, UK). This programmable robot can collect biomass from scintillation vials (www.zinsserna.com) for grinding, weighing, and transferring into 4 mL plastic vials (Micronic, USA) loaded into 48-vial plates. Biomass can be distributed across six plates in a single run to enhance throughput processing efficiency, while exhibiting a 10% weight error margin as shown in Fig 2. A 15% (w/v) biomass loading amount was established for the pretreatment reaction, resulting in approximately 0.225 g of biomass being transferred to each vial.

#### Pretreatment

The pretreatment step commences with the addition of IL to the biomass, facilitated by a liquid handling robot (BioMek NX 8, Beckman Coulter, USA) but also amenable to manual pipetting for smaller sample sets. The synthesis of the IL cholinium phosphate involved combining cholinium hydroxide and phosphoric acid in a 3:1 mole ratio through slow overnight titration on an ice bath and blending it with water to achieve a 5% (w/w) concentration. After IL transfer, the vials were securely capped using the Micronic screw cap recapper CS500 (Micronic, USA) and subjected to a 3 h treatment at 121°C and 15 psi in an autoclave (Yamato Scientific America). The choice of employing Micronic plates was informed by prior unsuccessful attempts with different plate types and heating methodologies (S1 Fig). The adoption of Micronic vials that are individually capped inside racks with an open bottom, facilitated uniform heat distribution and met our requirements for miniaturization and high-throughput sample processing (S2 Fig).

#### Enzymatic saccharification

The dilute cholinium phosphate aqueous solution exhibits an alkaline nature, with a pH exceeding 11. After biomass pretreatment, the pH of the pretreated biomass mixture was found to be close to 8. Saccharification of the biomass was facilitated by a blend of Cellic® CTec3 and Cellic® HTec3 enzymes (Novozymes, USA) at a ratio of 9:1 (v/v) and a concentration of 10 mg of enzyme per g of biomass. The enzymatic activities that effectively degrade polysaccharide chains manifest optimally at pH values close to 5 [18]. This poses a challenge in maintaining the pH of the pretreated biomass mixture within the enzymatically active range. This challenge was surmounted by the addition of citrate buffer (pH 5.0) concurrently with the enzyme mixture. The optimal volume of buffer was determined through iterative trials and finally set at 600 μL, resulting in a final buffer concentration of 0.4 M. Subsequently, the vials were securely recapped and the plates were incubated at 50°C in a hybridization oven equipped with a rotating platform affixed with securing straps. The vertical rotation at 30 rpm ensures thorough and uniform mixing of the pretreated biomass, enzymes, and buffer, facilitating saccharification over a 72-hour period. An automated robotic pH meter (Hudson Robotics, USA) was used to confirm pH values close to 5.3 for pretreated biomass samples after buffer addition in 48-well plates (S3 Fig).

#### Fermentation

The plates containing the saccharified hydrolysates were centrifuged using a bench scale centrifuge (Eppendorf 5810R) to separate the undigested biomass. The supernatants were then filtered using 0.45 μm filter plates with 1 mL sample capacity (Pall AcroPrep, USA). A small portion of the hydrolysate was used for sugar analysis using HPLC following a previously published protocol [19] and the remaining hydrolysate was conditioned for the fermentation process. The conditioning involves adjusting the pH to a value of 7 to enhance microbial growth by addition of NaOH and phosphate buffer. To keep the volumes constant across many different samples, 800 μL of the hydrolysate were mixed with 150 μL of 1 N NaOH and 150 μL of 0.5 M phosphate buffer (pH 7.0). Fermentation of sorghum hydrolysates was performed using a *Rhodosporidium toruloides* strain named GB2 that has been engineered to produce the aviation fuel precursor bisabolene [20]. The fermentation step involves cultivating the engineered *R*. *toruloides* on the conditioned and filtered hydrolysates using 48-well Flowerplates (m2p-labs, Germany) at 30°C and 900 rpm in a multitron orbital shaker (Infors HT, USA) for 7 days. Finally, samples were collected and analyzed by GC-MS to quantify bisabolene production.

#### Data processing

**Fig 3:**
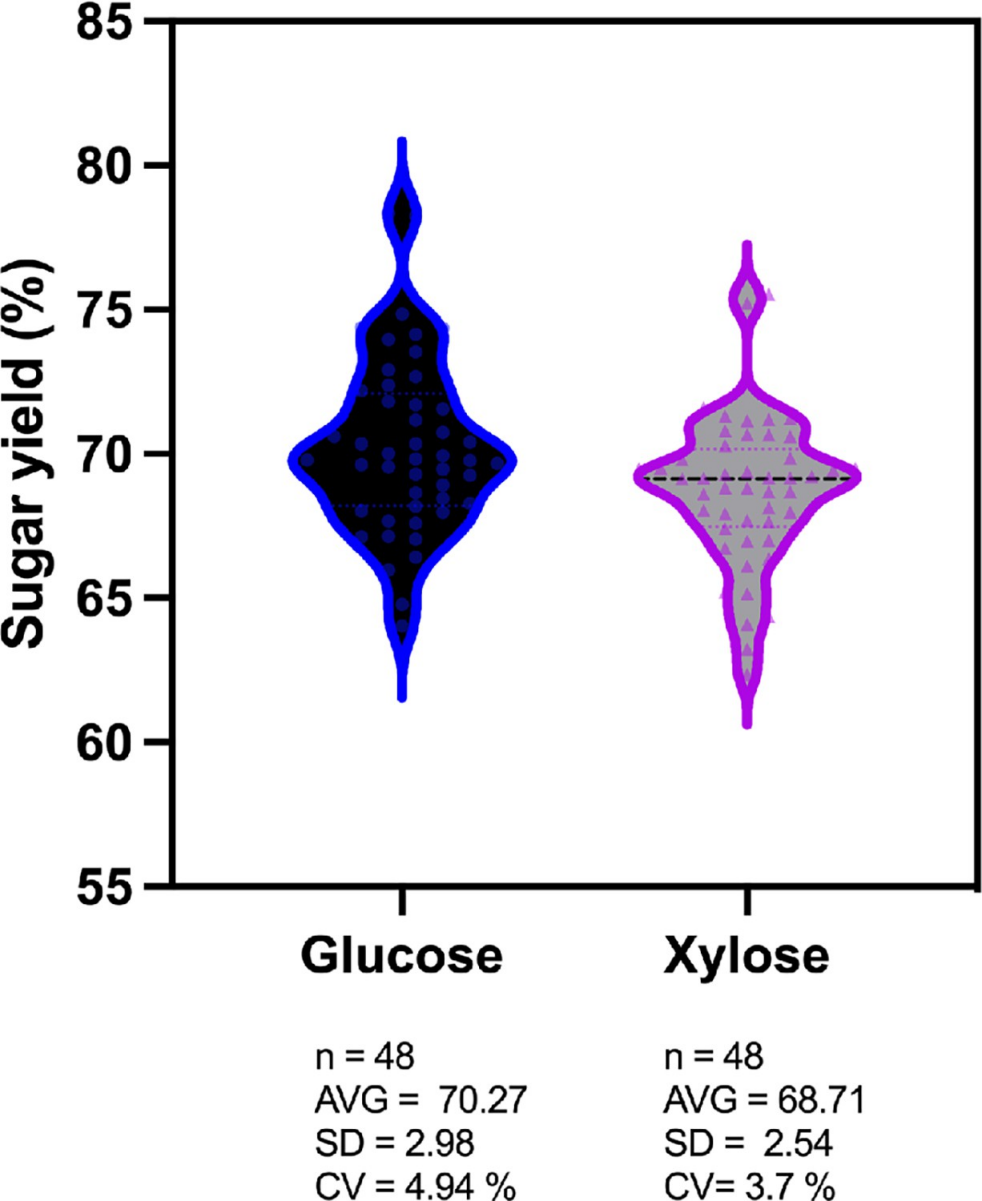
Glucose and xylose yields from wild-type sorghum biomass. The dots represent the 48 replicates. AVG = average; SD = standard deviation; CV = coefficient of variation.

**Fig 4:**
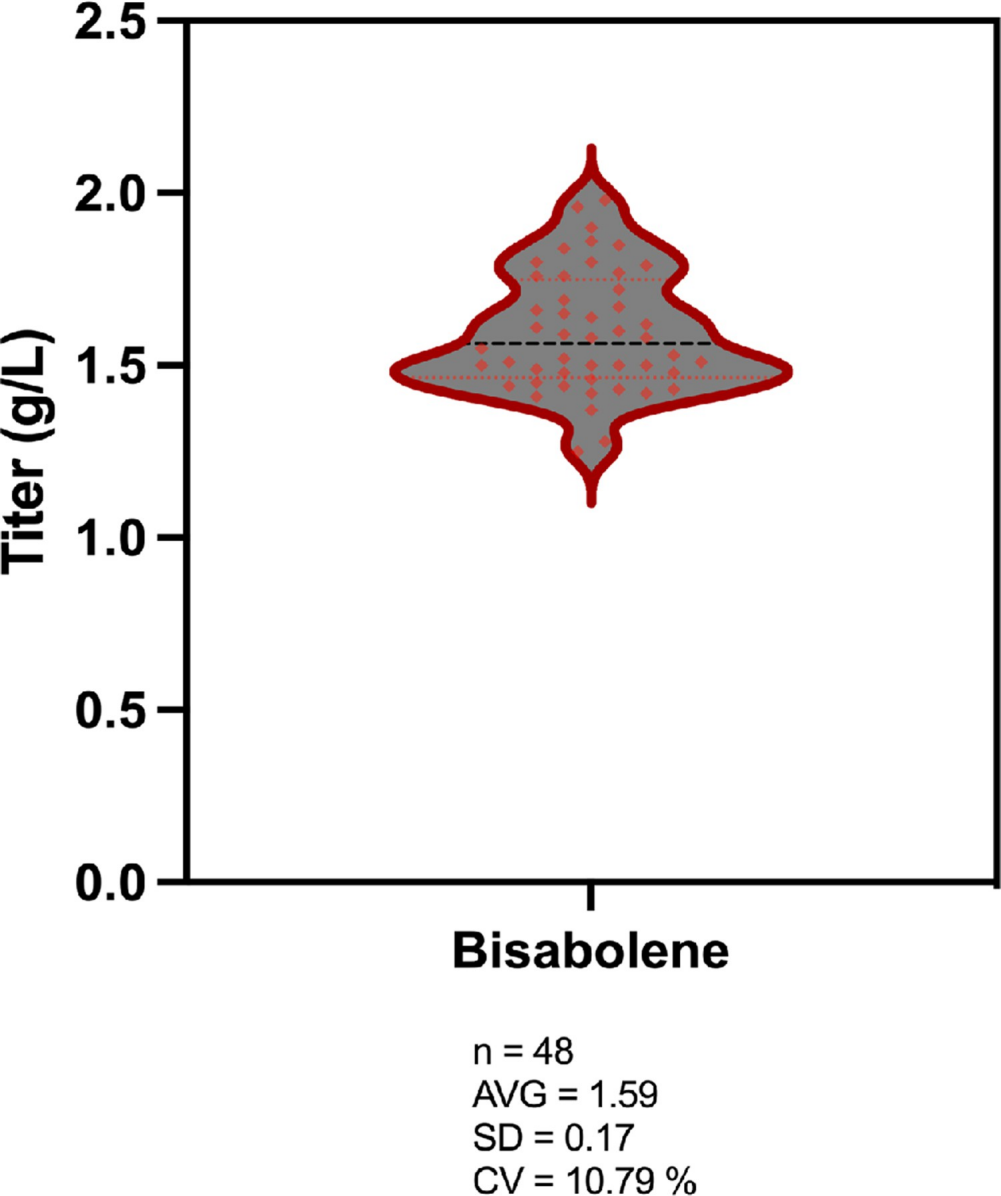
Bisabolene concentrations measured after 168 hours of fermentation in sorghum hydrolysates. The dots represent the 48 replicates. AVG = average; SD = standard deviation; CV = coefficient of variation.

The concentrations of glucose and xylose in hydrolysates were used to calculate conversion yields from biomass for all 48 samples (Figs 2 and 3). The average glucose yield was 70.27 ± 2.98% (standard deviation) with a coefficient of variation of 4.94%. The average xylose yield was 68.71 ± 2.54% (standard deviation) with a coefficient of variation of 3.70%. The average bisabolene titer was 1.59 ± 0.17 g/L (standard deviation) with a coefficient of variation of 10.79% (Fig 4). Although not strictly comparable since the reaction conditions are different, the obtained sugar yields and bisabolene titers were similar to those previously reported when using the related IL cholinium lysinate [19]. All results obtained by HPLC and GC-MS were transferred to the Experiment Data Depot (EDD), an online tool that serves as a repository of biologically interpretable data and provides data visualization and standardization [21, 22]. Integrating this tool with the F2F pipeline is crucial to ensure preservation of the data and metadata from large sample sets, decrease the time required to plot and analyze the data, and provide the capability to export them for use with predictive algorithms [23].

## Discussion

**Fig 5:**
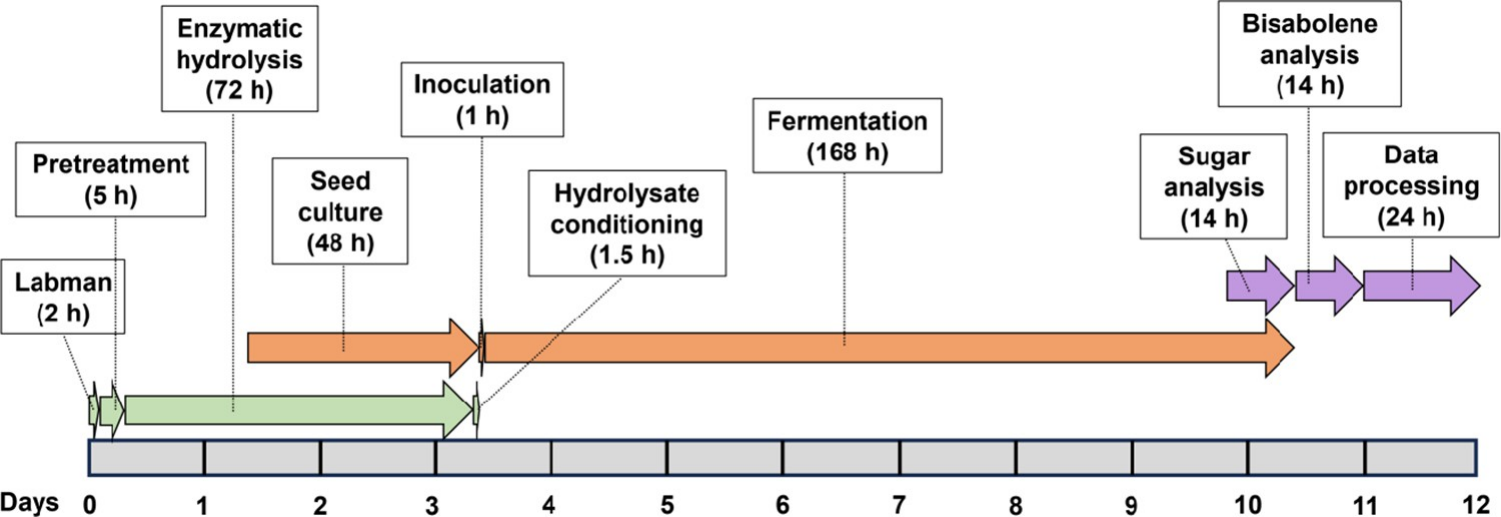
Scheme of the general steps and approximate times required for processing 48 samples with the F2F pipeline. The green, orange and purple arrows indicate the main tasks included in the deconstruction, fermentation, and analysis stages, respectively.

A key advantage of the generated pipeline is its scalability in terms of the number of samples that can be processed per unit of time. In Fig 5, we show a scheme of the steps and completion times required to process a set of 48 samples, which is approximately 12 days. Fermentation is the longest step (7 days) but since we employ multi-well plates that are stackable, a large number of samples can be simultaneously processed in a single incubator. Other steps like seed culturing and enzymatic hydrolysis or fermentation and sugar analysis can be performed in parallel. We estimate that the time needed to process 480 samples will be 25 days, a 10-fold throughput increase in only twice the amount of time compared to 48 samples. In this case, the Labman robot would need 20 additional hours, the pretreatment and fermentation times would remain the same, sample handling and preparation for analysis would require 10 additional hours, and bisabolene analysis would require 126 additional hours. In some cases, measuring the residual sugar concentration could be informative to calculate the microbial consumption percentage, which adds approximately 14 h of processing time for every set of 48 samples.

Notably, the pipeline described here can also be used to evaluate the effects of biomass loading, IL concentration, pretreatment temperature, and enzyme concentration on the saccharification yields, cell growth, and bisabolene titers. Some immediate applications include: 1) rapid screening of engineered feedstocks and novel pretreatment solvents and enzymes, 2) evaluation of hydrolysate biocompatibility and conversion efficiency by different engineered microbes in presence of ILs, 3) discovery of correlations of plant genotype and phenotype to deconstruction and conversion efficiency, 4) generation of datasets to feed computational models and enable predictive deconstruction and conversion.

## Supporting information

S1 File Step-by-step protocol, also available on protocols.io. (PDF)

S2 File Experimental data presented in this article. (XLSX)

S1 Fig Leakage, uneven heating, and drying of contents in conventional deep well plates. (PDF)

S2 Fig Micronic vials and biomass distribution. (PDF)

S3 Fig Automated pH meter to verify pH values of hydrolysates after enzymatic saccharification. (PDF)
