# Supplementary material for: A miniaturized feedstocks-to-fuels pipeline for screening the efficiency of deconstruction and microbial conversion of lignocellulosic biomass
Source: PLoS One. 2024 Oct 8;19(10):e0305336. doi: 10.1371/journal.pone.0305336 (PMC11460671; doi:10.1371/journal.pone.0305336)
Supplement: S1 File — (PDF) [file pone.0305336.s001.pdf]

Feb 17, 2024

Feedstocks-to-Fuels Pipeline (F2F)

Forked from a private protocol

DOI
dx.doi.org/10.17504/protocols.io.3byl4jrezlo5/v1

Venkataramana R Pidatala[1], Mengziang Lei[1], Hemant Choudhary[1], Christopher J Petzold[1], Hector Garcia Martin[1], Blake A Simmons[1], John Gladden[1], Alberto Rodriguez[1]

[1]Lawrence Berkeley National Laboratory

Venkataramana R Pidatala
Lawrence Berkeley National lab

OPEN ACCESS

DOI: dx.doi.org/10.17504/protocols.io.3byl4jrezlo5/v1

External link: https://sites.google.com/lbl.gov/esedataautomation/data-acquisition-storage/feedstocks-to-fuels-pipeline

Protocol Citation: Venkataramana R Pidatala, Mengziang Lei, Hemant Choudhary, Christopher J Petzold, Hector Garcia Martin, Blake A Simmons, John Gladden, Alberto Rodriguez 2024. Feedstocks-to-Fuels Pipeline (F2F). **protocols.io** https://dx.doi.org/10.17504/protocols.io.3byl4jrezlo5/v1

License: This is an open access protocol distributed under the terms of the **Creative Commons Attribution License**, which permits unrestricted use, distribution, and reproduction in any medium, provided the original author and source are credited

Protocol status: Working
We use this protocol and it's working

Created: February 07, 2023

Last Modified: February 17, 2024

Protocol Integer ID: 76617

Keywords: Feedstocks-to-Fuels pipeline, Biomass, Pretreatment, Enzyme Hydrolysis, Fermentation

Abstract

Sustainably grown biomass is a promising alternative to produce fuels and chemicals and reduce the dependency on fossil energy sources. However, the efficient conversion of lignocellulosic biomass into biofuels and bioproducts often requires extensive testing of components and reaction conditions used in the pretreatment, saccharification, and bioconversion steps. This restriction can result in an unwieldy number of combinations of biomass types, solvents, microbial strains, and operational parameters that need to be characterized, turning these efforts into a daunting and time-consuming task. Here we developed a high-throughput feedstocks-to-fuels screening platform to address these challenges. The result is a miniaturized semi-automated platform that leverages the capabilities of a solid handling robot, a liquid handling robot, analytical instruments, and a centralized data repository, adapted to operate as an ionic-liquid-based biomass conversion pipeline. The pipeline was tested by using sorghum as feedstock, the biocompatible ionic liquid cholinium phosphate as pretreatment solvent, a “one-pot” process configuration that does not require ionic liquid removal after pretreatment, and an engineered strain of the yeast Rhodosporidium toruloides that produces the jet-fuel precursor bisabolene as a conversion microbe.

Image Attribution

Venkataramana R Pidatala

Materials

Scintillation vials
4 mL Micronic vials
Cholinium hydroxide
Phosphoric acid
CTEC3:HTEC3 enzyme cocktail

Safety warnings

Wear proper PPE (gloves, safety goggle, and lab coat), and prepare solvents in a chemical fume hood.
Store organic solvents in a flammable storage cabinet when not in use.
Discard used solvents and buffers in appropriate waste containers.

Ionic liquid synthesis

1 Synthesize the ionic liquid (IL) cholinium phosphate by titrating cholinium hydroxide (Sigma-Aldrich CAS # 123-41-1) with phosphoric acid (Sigma-Aldrich CAS # 7664-38-2) at a 3:1 mole ratio inside a fume hood. Place cholinium hydroxide in a round bottom flask with a magnetic stirrer on an ice bath attached to a burette containing phosphoric acid. Use slow dripping to complete the reaction overnight. Lyophilize the IL for 3 days at -50 ºC to reach a semi-solid consistency.

Cholinium hydroxide and phosphoric acid come mixed with water and the percentage may vary based on the manufacturer. Calculate the mole ratio based on the specific amounts.

Biomass dispensing

2 Transfer milled biomass (1 mm particle size) to scintillation vials, filling approximately 75% of the vial.

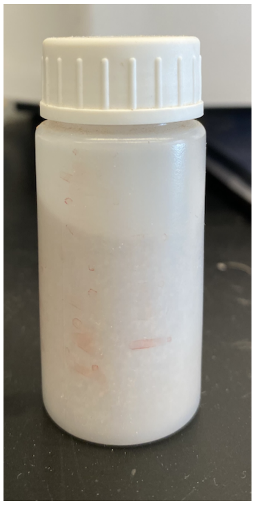

Fig. 1: Scintillation vial with milled biomass.

3 Dispense biomass from scintillation vials into Micronic vials using the Labman robot by following the protocol listed below.

For 15% biomass loading: Transfer 225 mg of ground biomass into 4 mL Micronic vials (in a 48 vial holder) using the Labman.

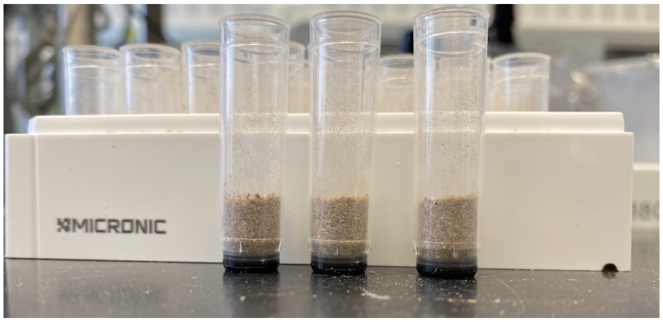

Fig. 2: 4 mL Micronic vials containing the dispensed biomass.

Protocol

NAME

Feedstocks-to-Fuels Labman solid biomass dispensing protocol

CREATED BY

Christopher J Petzold

PREVIEW

3.1 The graphical layout of this user interface (UI) is intended to resemble the deck of the Labman if you were facing the deck on the same side as the monitor. 1m

- If the software is already open when you start your run: click on the "**Clear Racks**" button of the "Function Buttons". This will clear out settings from the previous run
- If you opened the software yourself, click on the "**Initialize**" button of the "Function Buttons". This will allow all electronics to calibrate prior to the run.

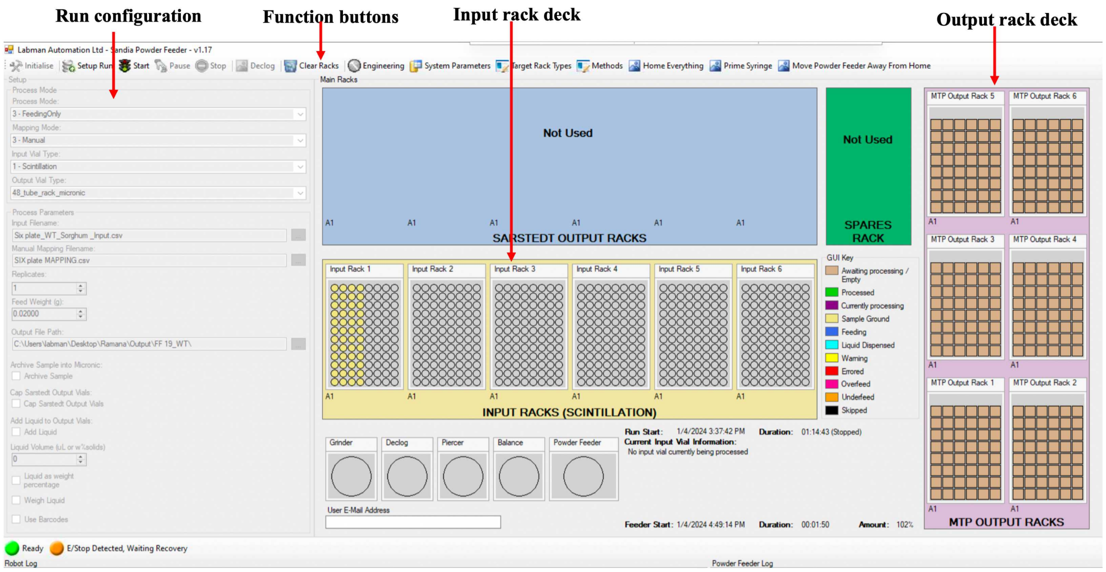

3.2 **Process Mode:** - Select "FeedingOnly" 1m

Mapping Mode - Select “Manual”

Input Vial Type - Select "Scintillation tube" or "Sarstedt tube" depending on the format of your samples

Output Vial Type - Select “Micronic 48”

3.3 **Process Parameters:**

Input Filename - Input csv file

Manual Mapping Filename - manual mapping csv file

Output File Path - Output file path

Input filename - The input file ('Input Filename' option) is a csv file that tells the robot where the source vials will be located. It has four columns.

A	B	C	D
Sample Description	Source Rack Number	Source Vial position	Method Name
1	1	1	200mg_sorghum_method
1	1	2	200mg_sorghum_method
1	1	3	200mg_sorghum_method
1	1	4	200mg_sorghum_method
1	1	5	200mg_sorghum_method
1	1	6	200mg_sorghum_method
1	1	7	200mg_sorghum_method
1	1	8	200mg_sorghum_method
1	1	9	200mg_sorghum_method
1	1	10	200mg_sorghum_method

A	B	C	D
1	1	11	200mg_sorghum_method
1	1	12	200mg_sorghum_method
1	1	13	200mg_sorghum_method
1	1	14	200mg_sorghum_method

Column A - Required, do not modify.
Column B - This column indicates the rack position for the source vials. These numbers are indicated on the deck.
Column C - This is the source well of the tube rack indicating where the source well is placed. 1 = A1 (bottom left corner), and 96 = H12 (top right corner).
Column D - This is the dispensing method that will be used by the Labman for distributing biomass.

3.4 **Manual Mapping filename** - The “Manual Mapping” file is a csv file that assigns a source (or input) vial to a destination (or output) vial. Note that these columns have labels, while the input file does not.

A	B	C	D	E	F
OutputRack	OutputRackPosition	InputRack	InputRackPosition	DispenseWeight (g)	LiquidVolume (uL)
1	1	1	1	0.2	0
1	2	1	1	0.2	0
1	3	1	1	0.2	0
1	4	1	1	0.2	0
1	5	1	1	0.2	0
1	6	1	1	0.2	0
1	7	1	1	0.2	0
1	8	1	2	0.2	0
1	9	1	2	0.2	0
1	10	1	2	0.2	0
1	11	1	2	0.2	0
1	12	1	2	0.2	0
1	13	1	2	0.2	0
1	14	1	2	0.2	0
1	15	1	3	0.2	0

Column A - OutputRack → This is the rack position where the destination plates will sit. For micronic48 tube racks, these will sit in the “MTP Output Rack” position on the deck (see Software Layout figure for details).

Column B - OutputRackPosition → This is the well in the tube rack that contains the destination tube used for this step. Numbering and position of wells is the same as for the input csv file (A1 = 1, F8 = 48, A1 is at bottom left hand corner if facing the robot).
Column C - InputRack → This is the deck position where the source tubes are located. This should be the same as the rack specified in "Column B" of the Input CSV file.
Column D - InputRackPosition → This is the well location of the rack where the source tube will be located. Note that numbering and layout convention is the same as used for the Input file CSV.
Column E - DispenseWeight → The weight (in g) of biomass to be transferred from the source tube to the destination tube.
Column F - LiquidVolume → Although we NEVER use liquid dispensing on the Labman, this column must be specified on the csv file, and should always be set to 0.

3.5 **Output file path** - Create a new file prior to each run with the date in the name.

Note

The output files specify information such as intended vs actual amount of biomass distributed, and if any error occurs. For every well completed, a new csv file is specified containing all completed wells. The address located here is where output files are specified.

3.6 Setting up the deck:

1. Place all source and destination tubes on the deck in the designated positions.
2. Once the deck is set, close the doors completely. They are all magnetic, and should click into place.
3. Then click the big green button - located next to the FRONT emergency stop button.

Note

- The doors have a pneumatic trigger that freezes all activity when opening. Thus, you might hear a "hiss" when you open the doors.
- If it is closed well, there should be another audible "hiss", indicating that the robot is ready to run.

3.7 Click the "**Setup Run**" button in the "Function Buttons" portion of the screen.

Note

If the specified csv files are correct, then the well positions containing source and destination tubes should be highlighted in a beige color.

3.8 Once this is complete, click the "**Start**" button of the "Function Buttons". You should see the following checkbox:

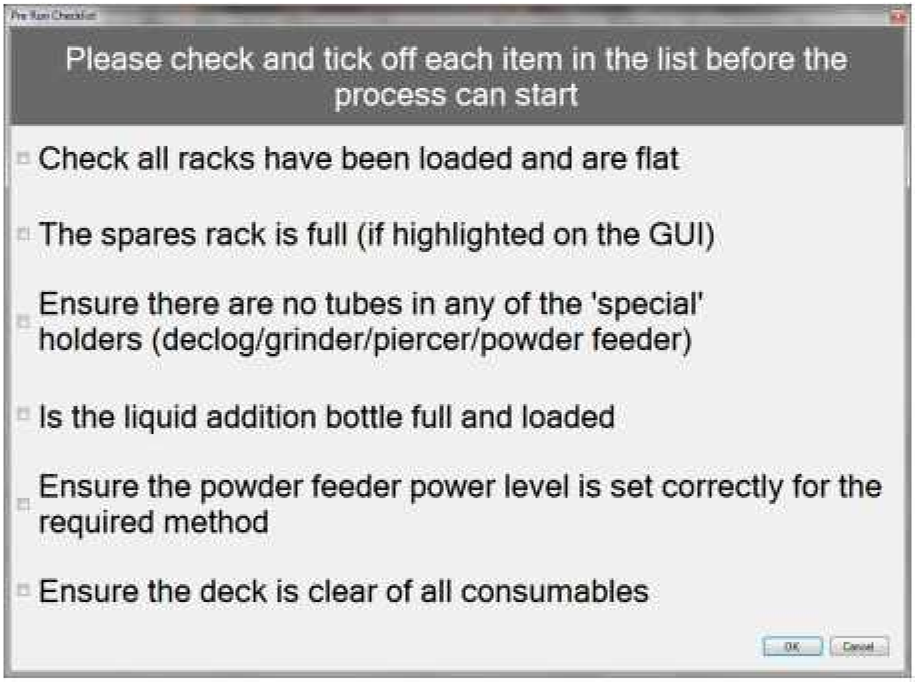

Note

This box simply exists as a reminder of safe operation to the user. All boxes must be checked prior to hitting OK even if that feature is not being used.

Clicking on 'OK' will initiate the run.

Note

It takes ~ 1 min to distribute ~200 mg of biomass into each micronic48 tube. This means that each full micronic plate will take ~ 1hr to complete a full plate.

Pretreatment

4 Prepare a mixture of 5% IL (w/w) and 80% (w/w) water and add the mixture to each vial by using a liquid handling robot or multichannel pipette.

Note

The volume of the IL and water mixture depends on the amount of biomass used in the experiment.

Example:
Biomass 0.225 g (15%) + IL (5%) + water (80%) mix: 1.275 mL.

5 Cap the vials and autoclave the plate with the vials at 121 °C for 03:00:00 . Remove the plate from the autoclave after cooling. 3h

Enzymatic hydrolysis

6 Measure the pH of the biomass after pretreatment and adjust it to a range of pH 4.8 - pH 5.2 using [M] 1 Molarity (M) citrate buffer, pH 5.0.

Note

Note: When using 0.225 g of biomass (15% solids loading) and 5% cholinium phosphate, approximately 600 µL of buffer are required, depending on the initial pH.

7 Prepare a mixture of CTec3:HTec3 enzymatic cocktails at a 9:1 volume ratio. Add the mixture to the pretreated biomass in each vial at a final ratio of 10 mg of enzyme per g of biomass. 1000 µL of enzyme mix contains 104 mg of enzyme. In this protocol, 20 µL of enzyme mix was used.

8 Incubate the plate at 50 °C for 72:00:00 . 3d

Sugar analysis

9 Centrifuge the plates in a benchtop plate centrifuge at 4000 rpm for 10 min to precipitate the solid fraction from the aqueous hydrolysate. Transfer 50 µL of hydrolysate from each vial to a 96-well 0.45 µm filter plate using a liquid handler or multichannel pipette. Place the filter plate on top of a collection plate and spin the plate in a benchtop centrifuge at 4000 rpm for 10 min.

10 Prepare samples for HPLC by diluting the filtered hydrolysates 20 times with water (10 µL sample + 190 µL water) in a Bio-Rad hard shell 96-well plate (Cat # HSP9601). Samples can also be stored at -20 °C until analysis.

11 Analyze the samples in an Agilent Inifinity 1260 HPLC equipped with an Aminex HPX-87H column, using 4 mM sulfuric acid as mobile phase, 0.6 mL/min flow rate, 60 ºC column temperature, 20 min run time and a refractive index detector kept at 35 ºC during analysis. Integrate the peak areas and compare to calibration curves made with pure glucose and xylose standards.

Fermentation

1w 2d

12 **pH adjustment**
To adjust the pH before microbial cultivation, mix 800 µL of the 0.45 µm filtered hydrolysate with 150 µL of 1 N NaOH and 150 µL of 0.5 M phosphate buffer pH 7.0.

12.1 Transfer 1000 µL of the pH-adjusted hydrolysate to a 0.2 µm filter plate, spin at 4000 rpm for 30 min and collect the filtrate in a sterile plate. Use the filtered hydrolysate for the fermentations.

> Note
>
> All subsequent steps in this section should be performed under sterile conditions.

13 **Growth and adaptation of *Rhodosporidium toruloides***
Streak *Rhodosporidium toruloides* strain GB2 on an agar plate prepared using yeast peptone dextrose (YPD) medium and the antibiotics cefotaxime (100 µg/mL) and nourseothricin (50 µg/mL), and incubate it for 2 days at 30 C.

13.1 Inoculate a colony from the plate in 2 mL of liquid YPD medium and grow at 30 °C for 48:00:00 at 200 rpm shaking speed. 2d

13.2 Adapt the cells to the hydrolysate by preparing a mixture of hydrolysate:YPD (50:50), inoculate with cells from the YPD culture, and grow for 2 days. Use this culture for inoculating the pure hydrolysates.

14 **Inoculation of hydrolysates**
Transfer 800 µL of the 0.2 µm filtered hydrolysates into individual wells of a 48-well flower plate (M2P-labs.com, Cat # M2P-MTP-48-OFF) using a multichannel pipette.

14.1 Prepare a 50X stock of filter-sterilized ammonium sulfate (250 g/L). Add ammonium sulfate to the hydrolysates to reach a final concentration of 5 g/L. Add cefotaxime 100 µg/mL (Sigma cat # C7039) to the hydrolysates.

14.2 Measure the OD 600nm of the adapted *R. toruloides* GB2 culture using a non-inoculated hydrolysate as blank. Calculate the volume of the culture required to have a starting O.D. of 0.5 and inoculate the hydrolysates.

14.3 Add 200 µL of Durasyn 164 (INEOS Oligomers, USA) to the hydrolysates as overlay (25% of the culture volume). Cover the plate with a gas permeable sealing film (M2P-labs.com, product # F-GPR48-10).

14.4 Incubate the plate at 30 °C and 900 rpm in a Multitron incubator (Infors HT, USA) with humidity control (>70% humidity) for 7 days.

14.5 Remove the plate from the incubator. Transfer all the contents into clean 1.4 mL micronic tubes. Allow the aqueous phase and Durasyn layers to separate by leaving on the bench for

about 15 min. Transfer 0.1 mL of the aqueous layer into 900 µL of water, measure the OD 600nm of the mixture to determine the microbial growth after cultivation.

14.6 Spin the plate containing the 1.4 mL tubes at 400 rpm for 10 min to pellet the cells and separate the aqueous and organic phases.

Bisabolene analysis

15 Mix 10 µL of the overlay with 990 µL of ethyl acetate with napthalene (5 mg/L) as internal standard in GC-MS vials.

16 Analyze samples on a GC-MS Intuvo 9000 instrument using the following method. Column: HP-5ms, inlet temperature: 250 °C, column initial temperature: 80 °C, ramp 20 °C per min until 300 ºC and hold for 3 min. Calculate bisabolene concentrations by integration of the peak area values and compare to the areas obtained from a calibration curve made with pure bisabolene.

Data processing

17 Compile results from sugar, bisabolene and OD 600 nm measurements and upload to the Experiment Data Depot following the instructions in the protocol below.

Protocol

EDD Data Import

NAME

Experiment Data Depot (EDD) Data Import

CREATED BY

Christopher J Petzold

PREVIEW
