# Supplementary figures and images for: A miniaturized feedstocks-to-fuels pipeline for screening the efficiency of deconstruction and microbial conversion of lignocellulosic biomass
Source: PLoS One. 2024 Oct 8;19(10):e0305336. doi: 10.1371/journal.pone.0305336 (PMC11460671; doi:10.1371/journal.pone.0305336)

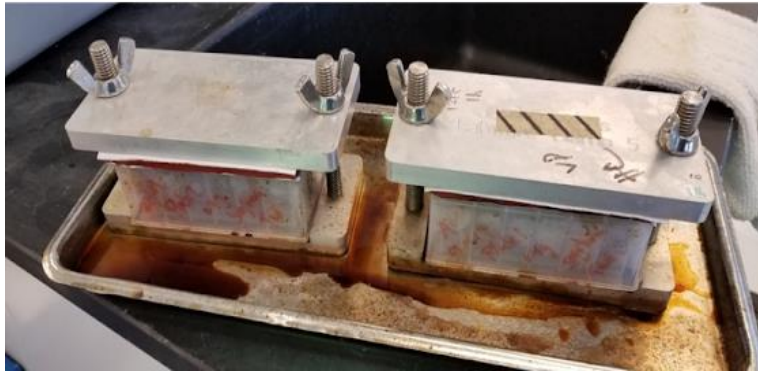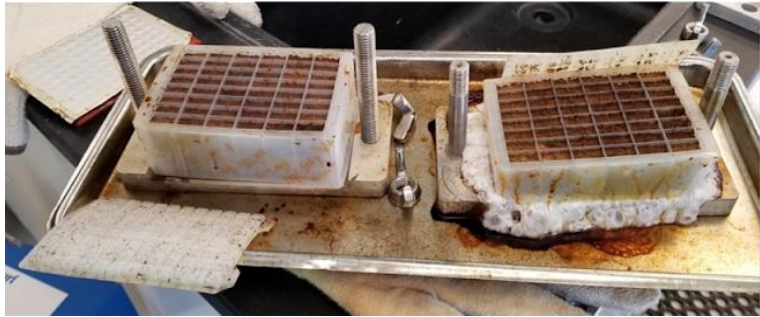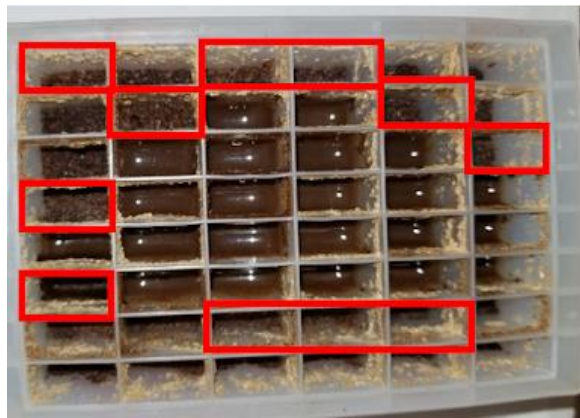

**S1 Fig.** Leakage, uneven heating, and drying of contents in conventional deep well plates.

Supplement: S1 Fig — (PDF) [file pone.0305336.s003.pdf]

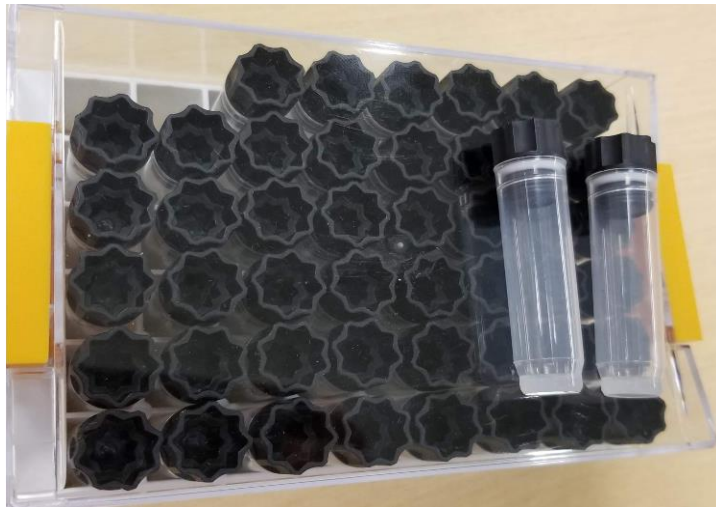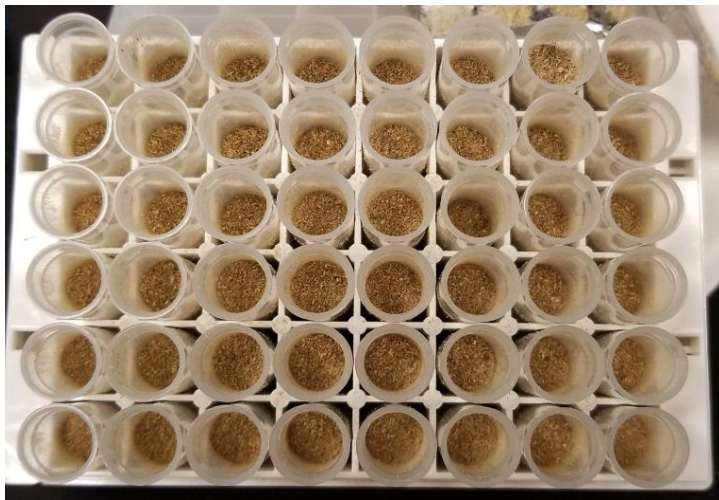

**S2 Fig.** Micronic vials and biomass distribution.

Supplement: S2 Fig — (PDF) [file pone.0305336.s004.pdf]
