# Supplementary material for: A miniaturized feedstocks-to-fuels pipeline for screening the efficiency of deconstruction and microbial conversion of lignocellulosic biomass
Source: PLoS One. 2024 Oct 8;19(10):e0305336. doi: 10.1371/journal.pone.0305336 (PMC11460671; doi:10.1371/journal.pone.0305336)
Supplement: S3 Fig — (PDF) [file pone.0305336.s005.pdf]

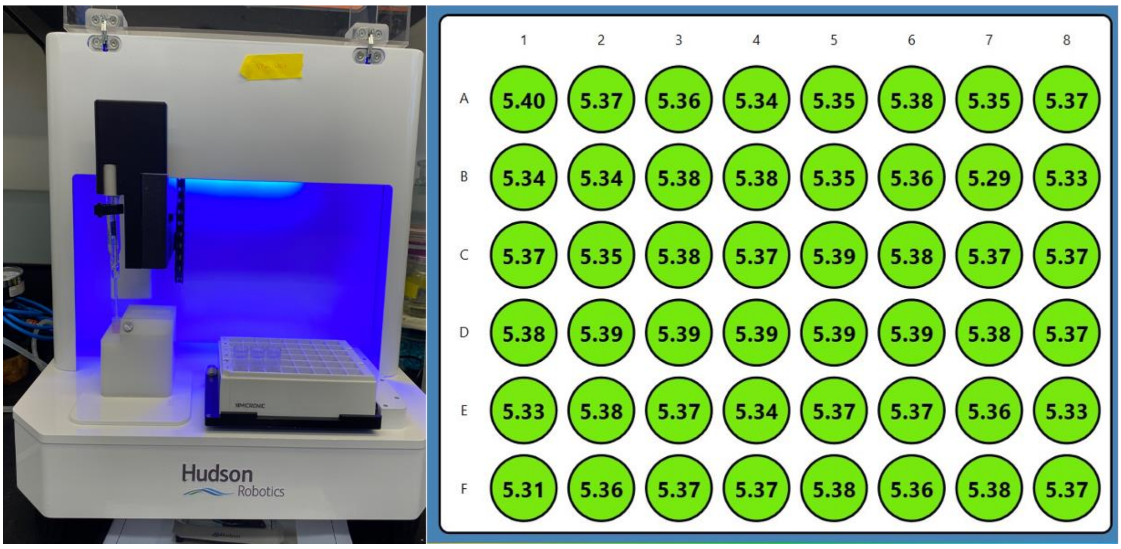

S3 Fig. Automated pH meter to verify pH values of hydrolysates after enzymatic saccharification.
